# Genomic links between symptoms of eating disorders and suicidal ideation

**DOI:** 10.1192/j.eurpsy.2025.25

**Published:** 2025-02-19

**Authors:** Agnieszka Musial, Una Foye, Saakshi Kakar, Tom Jewell, Janet Treasure, Gursharan Kalsi, Iona Smith, Laura Meldrum, Shannon Bristow, Ian Marsh, Chelsea Mika Malouf, Jahnavi Arora, Helena Davies, Rina Dutta, Ulrike Schmidt, Gerome Breen, Moritz Herle

**Affiliations:** 1 Social, Genetic & Developmental Psychiatry Centre, Institute of Psychiatry, Psychology & Neuroscience, King’s College London, London, United Kingdom; 2 Department of Mental Health Nursing, Florence Nightingale Faculty of Nursing, Midwifery & Palliative Care, King’s College London, London, United Kingdom; 3 United Kingdom National Institute for Health and Care Research (NIHR) Maudsley Biomedical Research Centre, South London and Maudsley NHS Foundation Trust, London, United Kingdom; 4 Department of Psychological Medicine, School of Academic Psychiatry, Institute of Psychiatry, Psychology & Neuroscience, King’s College London, London, United Kingdom

## Abstract

Eating disorders, including anorexia nervosa, bulimia nervosa and binge eating disorder, are psychiatric conditions associated with high mortality rates, particularly due to suicide. Although eating disorders are strongly associated with suicidal ideation, attempts, and fatalities, the precise relationship between these conditions remains poorly understood. While substantial genetic influences have been identified for both eating disorders and suicidality, the shared genetics contributing to their co-occurrence remain unclear. In this study, we utilized a multivariate approach to examine the shared genetic architecture of eating disorder symptoms, suicidal thoughts and behaviors in ~20,000 participants from the COVID-19 Psychiatry and Neurological Genetics (COPING) study. We applied individual-level structural equation modeling to explore the factor structure underlying eating disorder symptoms and suicidal ideation, followed by genetic correlation analyses. We modeled the general factor of susceptibility to eating disorders and suicidal ideation that was as strongly genetically influenced as both conditions, with mean SNP heritability of 9%. Importantly, despite the frequent co-occurrence of eating disorders with other psychiatric conditions, our findings highlight the specificity of the relationship between eating disorders and suicidality, independent of other co-occurring psychopathology, such as depression and anxiety. This specificity highlights the need for targeted approaches in understanding the shared susceptibility factors.

## Background

Eating disorders are characterized by one of the highest mortality rates among psychiatric illnesses, particularly among young individuals, with over 3.3 million premature deaths globally each year [1–4]. Anorexia nervosa (AN) affects from 0.3 to 1% of women in their lifetime and is a severe psychiatric condition marked by the inability to maintain a healthy body weight and poor prognosis [5, 6]. A comparatively large proportion of individuals with eating disorders die of suicide [3, 7], specifically individuals with AN (one in five deaths) [8].

Even though eating disorders have been associated with suicidal ideation, attempts and death, exactly how remains poorly understood. Eating disorder symptoms have been hypothesized to lead to suicidal thoughts and behaviors [9]. Conversely, suicidality may contribute to the development of eating disorders [9]. In addition, both eating disorders and suicidality may share underlying biological and psychological mechanisms, increasing lifetime susceptibility to both conditions [9]. Limited evidence exists for the influence of eating disorder symptoms on suicidality due to a lack of comprehensive longitudinal studies examining whether eating disorder factors predict later suicide outcomes [9]. A meta-analysis of 14 longitudinal studies revealed that eating disorders significantly predicted suicide attempts but were not found to be differentially predictive of death [10]. Further, eating disorder symptoms still accounted for individual differences in suicidality, although to a lesser extent, after controlling for their co-occurrence with other psychiatric disorders with increased risk of suicide, such as major depressive disorder [11–16]. There is a limited body of research exploring whether suicidality precedes the onset of eating disorders, with some studies reporting the onset of eating disorder symptoms following suicidal thoughts and attempts [17, 18]. Given the conflicting literature, a bidirectional causal relationship could be hypothesized.

Twin research has demonstrated substantial heritability, i.e., the degree to which individual differences in a trait can be attributed to genetic differences, of eating disorders, their symptoms, suicide and suicidal thoughts and behaviors [19]. A recent review [20] summarized the literature with heritabilities of 16–74% for AN, 28–83% for BN and 39–45% for BED. Similar estimates of genetic influences (30–55%) on suicidal behaviors were demonstrated by a subsequent large-scale systematic review of 32 studies [21]. A recent population-based twin study reported that genetic influences account for half of the variance in suicidal and self-harm behaviors, with 55% of the variation accounted for by genetic influences in non-suicidal self-harm and 50% in suicidal self-harm [22].

Complex psychiatric phenotypes, such as eating disorders and suicidal ideation and behaviors are highly polygenic, meaning that individual variation in these traits is influenced by a multitude of common genetic variants, with small effects [23, 24]. There have now been multiple genome-wide association studies (GWAS) studies of AN, but GWAS for other eating disorders and their symptoms are lacking [25]. The largest AN GWAS to date meta-analyzed data across 16,992 AN cases and found eight significant genetic regions/loci and estimated SNP heritability, the proportion of phenotypic differences accounted for by differences in common genetic variants, as ranging between 11 and 17% [26]. Nonetheless, the contribution of common SNPs to individual variation in suicidality differs depending on phenotype specification [27]. Based on a GWAS of nearly 40,000 cases reporting suicidal thoughts and behaviors, also encompassing self-harm and suicidal attempts, the SNP heritability was estimated as 7.6% [28]. The contribution of common variants to suicide attempts specifically have been estimated as ranging between 3.6 and 4.6% [27, 29, 30], with substantially higher estimates derived for completed suicide, ranging between 25 and 48% [31, 32].

Although the evidence suggests that symptoms of eating disorders and suicidality are substantially genetically influenced, little is known about their common genetic etiology, perhaps explaining their frequent co-occurrence. Family research exploring the shared liability of eating disorders, and suicide attempts suggested common familial and genetic factors influencing both outcomes [33]. Moderate-to-high genetic overlap between eating disorders and suicidality of 0.60 was demonstrated by twin studies for lifetime diagnosis of any eating disorder and suicidal thoughts [34] and 0.49 between lifetime AN diagnosis and suicide attempts [35]. In contrast, quantitatively assessed eating disorders and measures of suicidal and non-suicidal self-harm yielded weaker shared genetic etiology, with genetic correlations ranging between ~0.20 and ~ 0.40 [22]. Despite substantial shared genetic etiology indicated by family approaches, recent genome-wide approaches have found only a modest genetic correlation of 0.33 between AN and suicide attempts [36].

The majority of genome-wide analyses have focused on clinically assessed categorical phenotypes, limiting the ability to differentiate between variance common to a set of symptoms and variance specific to each. In the present study, we leverage a multivariate approach to examine the overlapping genetics of symptoms of specific eating disorders, including AN, BN, and BED and suicidal ideation. We explore the shared variance between these two broad constructs by investigating the latent structure underlying symptoms of eating disorders and suicidal ideation. By exploring these shared genetic components, we can gain a deeper understanding of the biological mechanisms underlying the co-occurrence between these conditions, as well as the degree to which symptoms of eating disorders and suicidal ideation are aetiologically unique.

## Methods

### Sample

The sample included participants from the National Institute for Health and Care Research (NIHR) BioResource who joined the COVID-19 Psychiatry and Neurological Genetics (COPING) study [37]. Alongside COVID-related measures, the COPING study incorporated questionnaires from the Genetic Links to Anxiety and Depression (GLAD) Study and the Eating Disorders Genetics Initiative UK (EDGI UK) [38, 39]. For further information on the sub-cohorts, recruitment and exclusion criteria, please refer to [37, 40]. For details on genotyping and quality control of the samples please refer to Supplementary Note 1. Our selected sample included a total 20,810 individuals from GLAD (*N* = 9,485), EDGI UK (*N* = 900) and NBR sub-cohorts of the COPING study (*N* = 10,425). The mean age of the sample was 49.3 years (*SD* = 17.56). Females comprised 71% (*N* = 14,673) of the sample and 97% (*N* = 20,114) of participants reported European ethnic origin.

Because the GLAD study recruited participants based on lifetime history of depression or anxiety, participants who had experienced these conditions constituted 59% (*N* = 12,337; 80% females) of the sample, with 3,639 individuals (74% females) diagnosed with major depressive disorder, 1,170 individuals (81% females) diagnosed with generalized anxiety disorder and 7,528 individuals (83% females) diagnosed with both conditions. EDGI UK recruited participants with a lifetime probable or clinical eating disorder, resulting in 8% (*N* = 1,748; 96% females) of the sample reporting being diagnosed with any eating disorder, of whom 810 individuals (96% females) had a lifetime diagnosis of AN, 275 individuals (99% females) a diagnosis of BN and 322 individuals (87% females) a diagnosis of BED. Further, 255 individuals (99% females) reported both AN and BN diagnoses over their lifetime, 56 individuals (96% females) reported AN and BED diagnoses and 110 (98% females) reported BN and BED diagnoses. In addition, 91 individuals reported being diagnosed with purging disorder, 157 with avoidant/restrictive food intake disorder, 13 with rumination disorder and 220 with other feeding eating disorder. With COPING comprising largely individuals with a lifetime history of eating disorders and mood disorders, the clinical nature of the sample makes it generalizable to the clinical population of individuals with full threshold eating disorders.

### Measures

#### Eating disorders

Symptoms of eating disorders were assessed using the ED100K questionnaire that measures the severity and duration of lifetime eating disorder symptoms [41]. The ED100k questionnaire included Likert-scale items, as well as binary items, such as *During eating binges, did you feel ashamed/disgusted with yourself, depressed, or very guilty after overeating?*, which were summed up to create disorder-specific quantitative symptom scores related to weight and shape control, compensatory behaviors, excessive exercise and bingeing emotions/behaviors, where higher scores reflected more severe symptoms of eating disorders. For the current analysis, only items directly measuring eating disorder symptoms were retained, discarding items focusing on body measurements and duration of symptoms. We created symptom scores specific to AN, BN and BED by summing the items, resulting in higher symptom scores reflecting more/more severe symptoms of AN, BN and BED. For details on symptom scores and items included please refer to Supplementary Table 1.

### Suicidal ideation

Suicidal ideation was measured at COVID baseline using the following three items from the thoughts and feelings questionnaire (TAF) [42]: *Many people have thoughts that life is not worth living. Have you felt that way?*, *Have you contemplated harming yourself?* and *Before the pandemic, had you deliberately harmed yourself, whether or not you meant to end your life?* The remaining items temporally related to the COVID-19 pandemic were discarded.

### Psychopathology

Depressive symptoms were measured using an adapted version of the Patient Health Questionnaire-9 (PHQ-9) [43], which is a concise and validated tool used to assess the severity of depression. In the current paper we have dropped the *Thinking about how you usually felt before the pandemic, how much were you bothered by the thoughts that you would be better off dead or of hurting yourself in some way?* item from the PHQ-9, resulting in an 8-item measure. Symptoms of anxiety were assessed using the Generalized Anxiety Disorder-7 (GAD-7) [44]. The PHQ-9 and GAD-7 were administered during the sign-up surveys of the GLAD and EDGI UK and baseline COVID survey for other COPING sub-cohorts.

### Mental health diagnoses

Diagnoses of eating disorders, major depressive disorder and generalized anxiety disorder were evaluated based on the Mental Health Diagnosis questionnaire (MHD), adapted from the UK Biobank Questionnaire [45]. This questionnaire was integrated into the sign-up surveys of the GLAD and EDGI UK and baseline COVID assessment for the remaining COPING participants.

### Analyses

Analyses for this project were preregistered with the Open Science Framework (OSF).

(https://osf.io/csva6/; Supplementary Note 2). Scripts are available on https://github.com/agmusial/genomic_links_eds_su. All variables were residualised on participant age, sex, genotyping batch and 10 principal components of ancestry.

### Exploratory factor analysis

We performed an exploratory factor analysis (EFA) on AN, BN and BED symptom scores and the TAF items related to self-harm and suicidal ideation to determine the underlying phenotypic factor structure. Exploratory factor analyses were conducted in *psych* for R [46, 47], using 70% of the available data. The remaining 30% of the data was used to run the confirmatory factor analysis (CFA). Sensitivity analyses were performed on a smaller proportion of the sample, excluding individuals diagnosed with major depressive disorder and generalized anxiety disorder, resulting in a reduced sample size of 8,404 individuals, as well as sex-specific sub-samples of 6,135 male and 14,673 female participants. Models showing good fit with the data were then fitted on the genome-wide level via extracting factor scores and using them as phenotypes.

### Theoretical models

In addition to the data driven latent structure derived from the EFA and CFA analyses, we also tested a series of theoretical structures potentially underlying the co-occurrence between eating disorder symptoms and suicidal ideation. We tested conceptual models addressing latent structure of the co-occurrence between eating disorder symptomatology and suicidality, as well as distinguishing between restricting, purging and bingeing eating disorder subtypes. We fitted multiple iterations of the following three structures, including a hierarchical model, residual model and four-factor models (Supplementary Figure 1).

1. A hierarchical model included 6 observed variables (here, AN, BN, BED symptom scores and three suicidality items from the TAF questionnaire), loading onto first-order factors (here, eating disorders and suicidal ideation), which in turn loaded onto a second-order general susceptibility factor, underlying their co-occurrence (Supplementary Figure 1a).
2. A general susceptibility factor for eating disorder and suicidal ideation, allowing for independent domain specific variances. This residual model included a first-order general susceptibility factor, indexed by the manifest variables of eating disorder symptom scores and TAF items and specific factors of eating disorders and suicidal ideation that account for the residual variance in eating disorders and suicidality (Supplementary Figure 1b).
3. Differentiation between restricting, purging and bingeing eating disorder symptoms and their joint association with suicidal ideation. The four-factor model included four first-order factors (here, restricting, purging, bingeing and suicidal ideation), which were correlated (Supplementary Figure 1c).

Because mood disorders have been well documented to contribute to the risk of suicide, we tested each model with and without additional measures of depression and anxiety, replacing the general factor of eating disorders with general factor of psychopathology, as well as including an additional separate factor of psychopathology [11–16] (Supplementary Figure 2). All theoretical models were specified using sem (structural equation modeling) in *lavaan* for R with, incorporating the full information maximum likelihood (FIML) to mitigate data missingness [48–50].

### Genome-wide analyses

Following imputation and quality control (Supplementary Note 1), the resulting sample of 15,009,228 SNPs was used in GWAS. The GWAS were conducted in plink 2.0 [51] using factor scores extracted from previously fitted sem models as phenotypes, including the EFA-based factors of eating disorders and suicidal ideation, the theoretical general factor of susceptibility to eating disorders and suicidal ideation and residual factors indexing unique variance in both traits from the residual model, as well as latent factors of restricting, purging, bingeing and suicidal ideation from theoretical four-factor model. GWAS were followed by analyses of SNP heritability and genetic correlations between the extracted factors, using individual-level genotype data within GCTA-GREML (genome-wide complex trait analysis-genome-based restricted maximum likelihood) [52–54].

## Results

### Exploratory and confirmatory factor analyses

**Figure 1.**
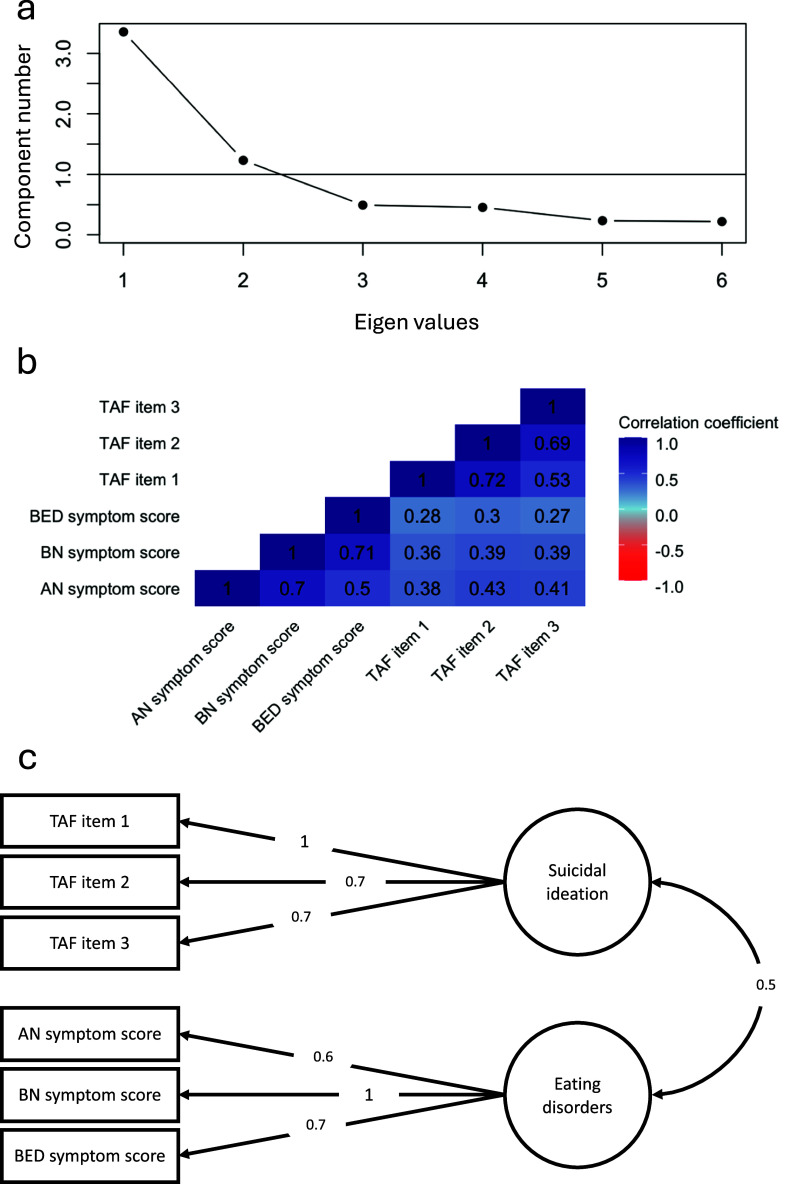
Results of the confirmatory factor analysis of AN, BN and BED symptom scores and suicidality items (*N* = 6,378). The figure depicts a two-factor model, where AN, BN and BED symptom scores load onto a factor of eating disorders and TAF items load onto a factor of suicidal ideation. The factors are correlated at r = 0.5. *Note.* AN = anorexia nervosa; BN = bulimia nervosa; BED = binge-eating disorder; TAF = thoughts and feelings questionnaire; TAF item 1 = *Have you contemplated harming yourself?*; TAF item 2 = *Many people have thoughts that life is not worth living. Have you felt that way?*; TAF item 3 = *Before the pandemic, had you deliberately harmed yourself, whether or not you meant to end your life?*

The EFA of AN, BN and BED symptom scores and TAF items revealed a two-factor structure (Figure 1 & Supplementary Figure 3), with eating disorder symptom scores loading onto a general factor of eating disorders and TAF items loading onto a general factor of suicidal ideation, which were moderately correlated at *r* = 0.5. The subsequent EFA that included additional measures of depression and anxiety yielded an equivalent 2-factor structure, with psychopathology measures loading onto the previously identified general factor of suicidal ideation (Supplementary Figure 3). Confirmatory factor analyses revealed substantial differences in model fit, with the two-factor model including psychopathology measures resulting in markedly worse fit compared to the model only including eating disorders, based on the difference in RMSEA statistics of 0.19, compared to 0.07. Including a separate third factor of psychopathology resulted in RMSEA of 0.07. Complete set of model fit indices is presented in Supplementary Table 2.

### Theoretical models

**Figure 2.**
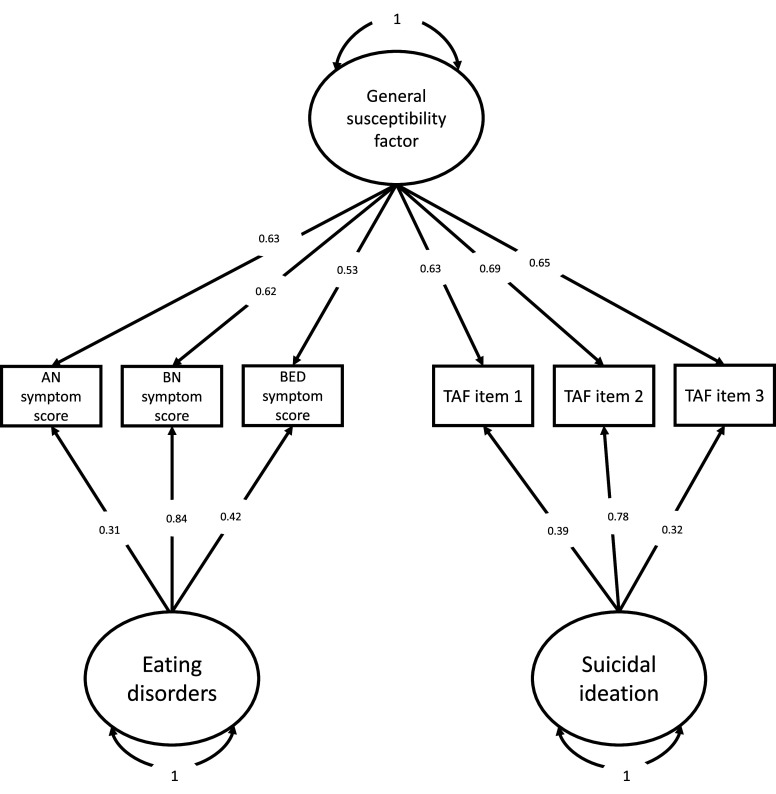
The residual model of a general factor indexing the co-occurrence between symptoms of eating disorders and suicidal ideation (N = 32,065). In this model, eating disorder symptom scores and TAF items load onto a higher-order factor of general susceptibility to eating disorders and suicidal ideation, capturing the shared variance between these conditions. Their unique (residual) variance is indexed by the residual factors of eating disorders and suicidal ideation. *Note.* AN = anorexia nervosa; BN = bulimia nervosa; BED = binge-eating disorder; TAF = thoughts and feelings questionnaire; TAF item 1 = *Have you contemplated harming yourself?*; TAF item 2 = *Many people have thoughts that life is not worth living. Have you felt that way?*; TAF item 3 = *Before the pandemic, had you deliberately harmed yourself, whether or not you meant to end your life?*

**Figure 3.**
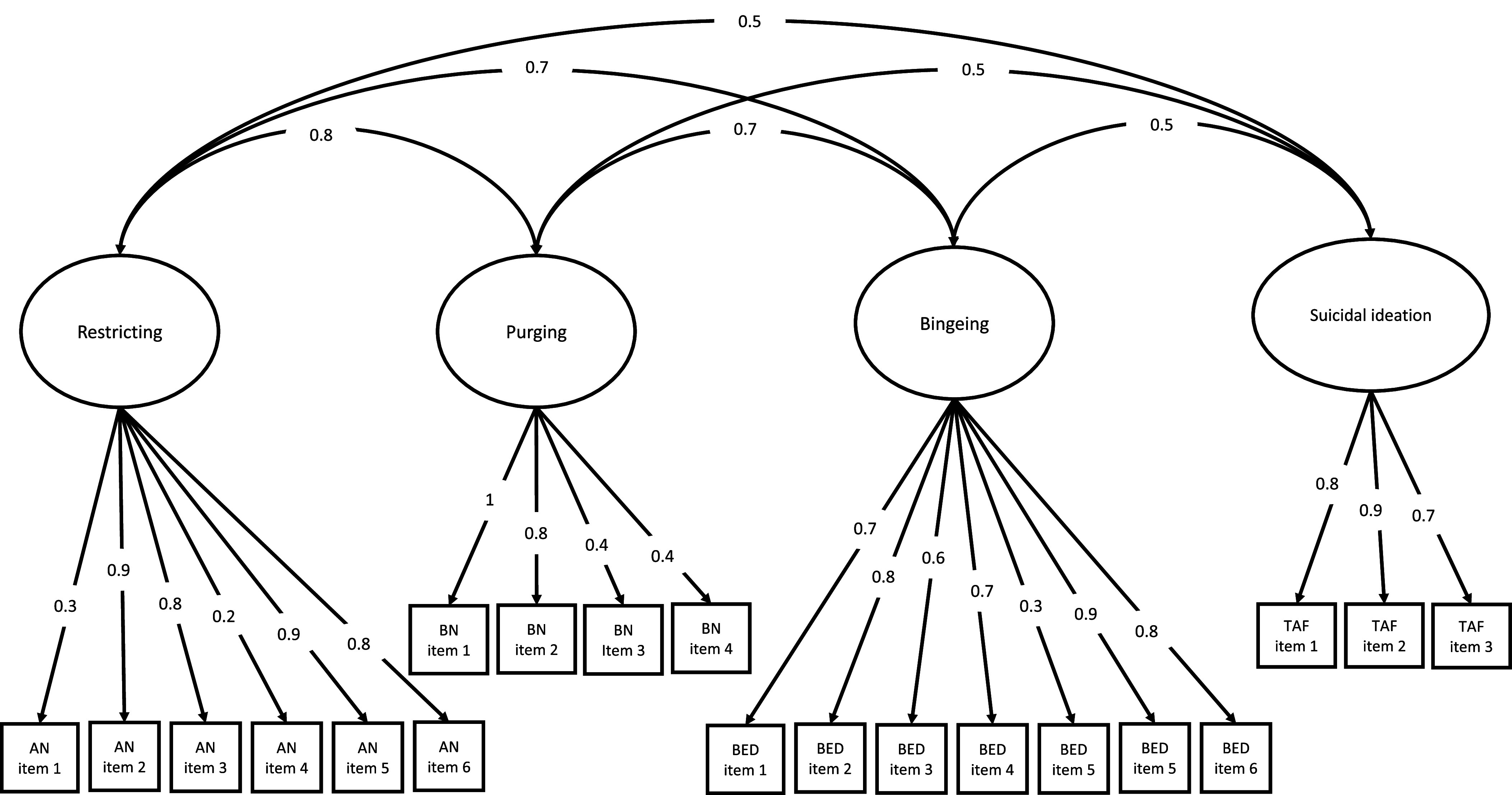
The four-factor model of restricting, purging, bingeing and suicidal ideation. In this model, AN, BN, BED symptom scores and TAF items respectively load onto factors of restricting, purging, bingeing and suicidal ideation, which are correlated (*N* = 32,065). *Note.* ED = eating disorder; AN = AN; BN = bulimia; BED = binge-eating; TAF = thoughts and feelings questionnaire; TAF item 1 = *Have you contemplated harming yourself?*; TAF item 2 = *Many people have thoughts that life is not worth living. Have you felt that way?*; TAF item 3 = *Before the pandemic, had you deliberately harmed yourself, whether or not you meant to end your life?.* Items are listed in Supplementary Table 1.

Among the theoretical models fitted, best fit was achieved by the residual model of a general factor indexed by eating disorder symptom scores and TAF items (Figure 2) and the four-factor model of restricting, purging, bingeing and suicidal ideation (Figure 3), with the RMSEA = 0.03 for both models. Including measures of depression and anxiety worsened the residual model fit to RMSEA of 0.04. Poor fit of RMSEA = 0.08 was yielded by the hierarchical model of two first-order factors of eating disorders and suicidal ideation and a second-order factor of general susceptibility underlying their co-occurrence and including psychopathology measures again resulted in further worsened fit of RMSEA = 0.19. The complete set of model fit indices across all iterations of theoretical models is presented in Supplementary Table 2.

### Genome-wide analyses

**Figure 4.**
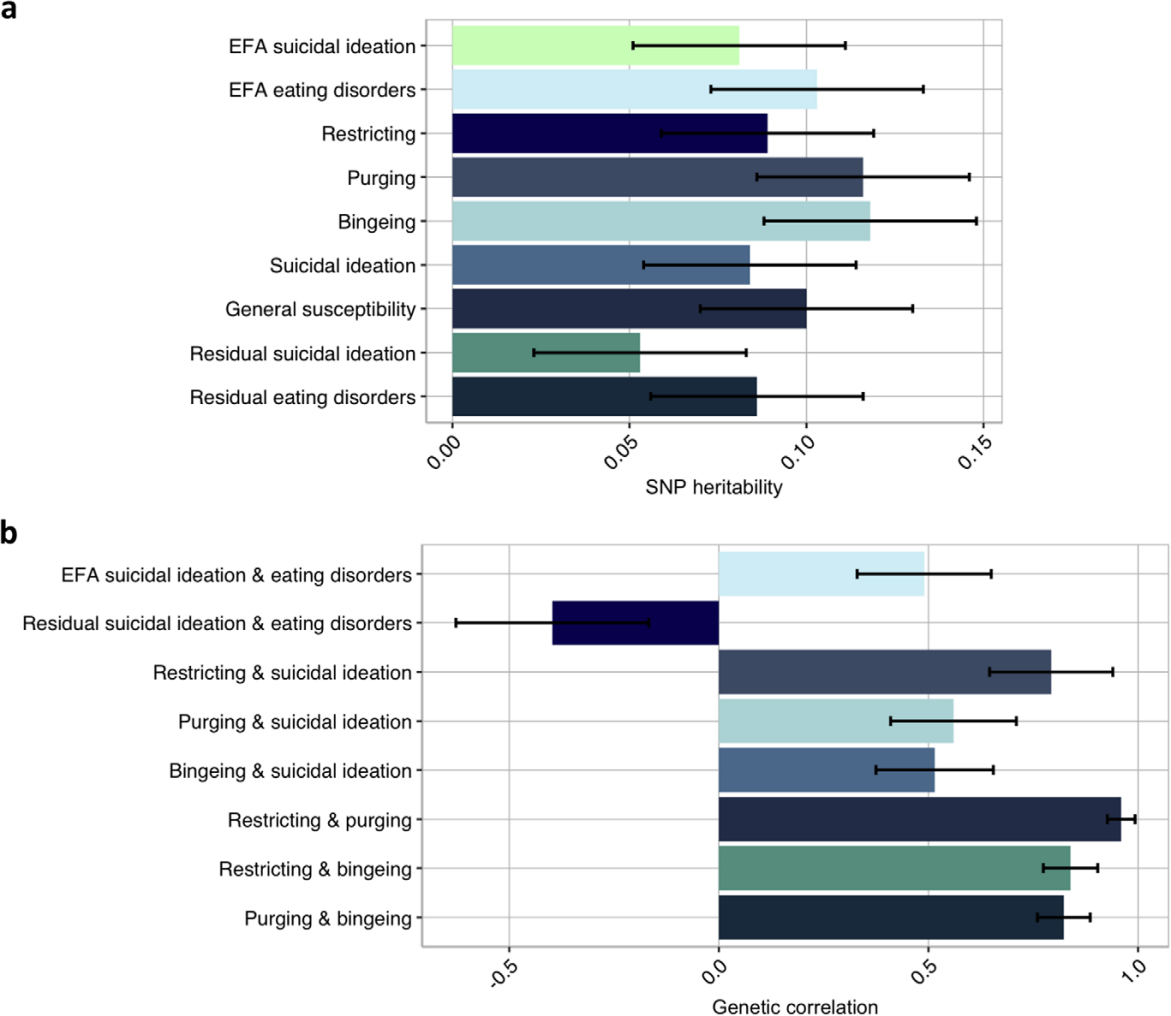
SNP heritability (panel a) of extracted factor scores from the EFA-based and theoretical models and genetic correlations between the factors (panel b) as estimated by the genome-wide complex trait analysis (GCTA). Error bars signify standard errors. *Note.* EFA = exploratory factor analysis.

Results of the GWAS analyses are illustrated in Supplementary Figure 4. As estimated using individual-level genotypes [53, 54], SNP heritabilities of the factors of suicidal ideation and eating disorders were modest, but significant with a mean SNP h^2^ of 0.09, ranging between 0.05 (0.03) for the residual factor of suicidal ideation and 0.12 (0.03) for the factors of purging and bingeing (Figure 4). The estimates did not differ significantly from one another. Factors of susceptibility to suicidal ideation and eating disorders were strongly positively genetically correlated across latent structures, with the mean genetic correlation of 0.71, while the residual factors of suicidal ideation and eating disorders were negatively correlated at −0.40 (0.23). Latent factors of restricting, purging and bingeing were genetically equivalent, with the genetic correlations ranging between 0.82 (0.06) and 0.93 (0.03). All estimates and standard errors are presented in Supplementary Table 3.

### Sensitivity analyses

The patterns of results of sensitivity analyses were equivalent to those obtained for the total sample, with best fitting models being the residual and hierarchical models. Estimates of SNP heritability and genetic correlations were similar for males and females, although the degree of precision was compromised due to reduced sample size. Full results of sensitivity analyses are presented in Supplementary Tables 4–6.

## Conclusions

Our study aimed to elucidate the phenotypic and genetic associations between eating disorder symptoms and suicidal ideation using a multivariate approach. On a phenotypic level, we identified a common latent factor contributing to susceptibility to eating disorders and suicidal ideation, both of which also presented substantial proportions of independent variance. These findings suggest a moderate degree of shared genetic architecture, supporting the hypothesis that these conditions are partially influenced by overlapping genetic factors. The exploratory and confirmatory factor analyses indicated a two-factor structure comprising distinct but correlated factors for eating disorders and suicidal ideation. This structure persisted even after accounting for additional measures of depression and anxiety. Among the various theoretical models tested, the residual model provided the best fit. This model posits a general susceptibility factor influencing risk to both eating disorders and suicidal ideation. The poor fit of models including measures of depression and anxiety highlights the specificity of the eating disorder-suicidality relationship, independent of co-occurring psychopathology, in contrast to literature suggesting a primary role of depression and anxiety in suicidality [11–16].

This general susceptibility factor likely represents the underlying biological or psychological mechanisms that contribute to a broad vulnerability to both eating disorders and suicidal ideation. Characterizing the markers acting as the common risk for eating disorders and suicidal ideation requires integrating genetic, neurobiological, and psychological perspectives. For instance, cytokine disruption, along with suboptimal nutritional status have been proposed to contribute to vulnerability to both conditions, though their predictive power remained modest [9, 54, 55]. Identifying endophenotypes or intermediate phenotypes, such as neuroimaging markers could help in understanding shared neurocognitive deficits [56, 57]. Exploring how environmental factors influence the development of both eating disorders and suicidal ideation could involve examining the role of emotion regulation deficits [58, 59], early life stress [60, 61] and trauma [62–64].

As mentioned above, we failed to support a substantial role of co-occurring psychopathology in the association between eating disorders and suicidal ideation. Research indicates that the majority of individuals who die by suicide have at least one psychiatric disorder at the time of death [65], however including measures of anxiety and depression in our phenotypic models resulted in markedly worse model fit as compared to models involving only eating disorder and suicidality measures. Hence, we did not support the previous findings of suicidality in individuals experiencing symptoms of eating disorder being solely a function of co-occurring mental health problems [66–69]. Poor fit of the models involving psychopathology measures persisted after excluding individuals diagnosed with major depression and generalized anxiety disorder, which is consistent with finding related to AN being associated with increased risk for suicidality, even after adjusting for psychiatric co-occurrence [68, 69]. Because affective disorders are highly prevalent among individuals with eating disorders and suicidal ideation there is a substantial overlap in their variance. Including measures of depression and anxiety in the models might have introduced multicollinearity or redundant information, potentially diluting the unique contributions of eating disorder symptoms to suicidality. This statistical redundancy may explain the poorer model fit when these variables were added.

The relationship between eating disorders and suicidal ideation appears to be highly specific, transcending the influence of co-occurring psychopathology. This specificity may stem from unique biological mechanisms shared between these conditions, including dysregulated neurotransmitter systems [67] and malnutrition that exacerbates brain-region dysfunctions critical for mood regulation [68]. Additionally, behaviors such as hopelessness about recovery and impulsivity [69, 70] may uniquely predispose individuals with eating disorders to experience suicidal thoughts, regardless of the presence of broader psychiatric symptoms. Therefore, future research should prioritize longitudinal studies to track the temporal interplay between symptoms of eating disorders and suicidal ideation, exploring whether one condition precipitates the other or if they emerge concurrently from shared vulnerabilities.

The genome-wide analyses demonstrated that the general factor of susceptibility to eating disorders and suicidal ideation, as well as the residual factors indexing unique variance in these traits, are significantly genetically influenced, with a mean SNP heritability of 8%. The residual factors were moderately genetically correlated (rG = −0.40). This might suggest that once the general genetic susceptibility is accounted for, the remaining variance for eating disorders and suicidal ideation are negatively related to each other. This negative correlation might reflect a compensatory or protective mechanism where the expression of genetic factors influencing one trait mitigates the risk of developing the other trait. For example, genetic variations that predispose an individual to eating disorders might simultaneously confer a lower risk for suicidal ideation, once the general susceptibility is controlled for. This negative relationship between symptoms of eating disorders and suicidal ideation should be interpreted with caution, as in the residual model the factors have been constrained to correlate through the general susceptibility factor and were otherwise set as orthogonal. Conducting longitudinal studies to track the development of eating disorders and suicidal ideation over time in individuals with elevated genetic predisposition for the general susceptibility factor could help in understanding the temporal dynamics of their relationship. Investigating the compensatory mechanisms could lead to new insights into resilience and potential protective factors that reduce the risk of eating disorders and suicidal ideation. However, this approach requires larger GWAS samples that would allow for identification and functional annotation of pleiotropic SNPs associated with the covariance between these traits.

While it has been indicated that a BN diagnosis alone does not predict mortality [8], we found that factors indexing restricting and bingeing/purging symptoms of eating disorders are strongly genetically correlated with suicidal ideation. These sub-types were also found to be equivalent on the genomic level, with the genetic overlap estimate of 0.98. While AN has traditionally been associated with higher suicide risk, BN and BED also exhibit similarly strong genetic correlations with suicidal ideation (genetic correlations of 0.79 and 0.64, respectively), emphasizing that various behaviors across symptoms of different eating disorders can predispose individuals to suicidal thoughts. While AN has received more attention in relation to suicide risk, it is important to adopt an inclusive approach in assessing and addressing suicide risk across individuals experiencing different types of eating disorder symptoms in clinical practice and research.

Several limitations must be acknowledged. While the sample size of over 20,000 participants is substantial considering phenotypic structural equation modeling analyses, GWAS analyses require larger samples to detect meaningful SNP associations, allowing for functional annotation and investigation of biological correlates of the identified latent structures underlying shared variance and estimation of significant genetic correlations between the constructs, where for the genetic correlation of 0.50 to be detected, and for average SNP heritabilities of 7% for both traits, our sample provided only 7% of power [70]. This issue was pronounced especially when the sample size substantially dropped following exclusion of individuals diagnosed with major depression or generalized anxiety disorder, leading to the bivariate models not converging. Furthermore, the cross-sectional nature of the study limits causal inference. While self-harm has previously been suggested to precede bingeing and purging behaviors [71], longitudinal studies are necessary to establish temporal relationships between eating disorder symptoms and suicidal ideation, clarifying whether disordered eating precedes or follows the onset of suicidal thoughts and behaviors and identify environmental and psychosocial factors that mediate their longitudinal relationship.

It has to be acknowledged that participants included in the study were recruited through specific research initiatives and bioresource centres, potentially introducing selection bias. Because our study predominantly included participants from the National Institute for Health and Care Research (NIHR) BioResource, particularly those involved in the GLAD study, which is skewed towards individuals who have a predisposition or are actively managing anxiety and depression, our findings may not fully generalize to the broader population, especially those without pre-existing mental health conditions or those not actively engaged in mental health studies. In addition, it should be acknowledged that the Coping cohort is largely of European ethnic background and the reported genome-wide association results are likely to not be generalizable in other ancestral populations [72].

Our findings on the shared genetic underpinnings between symptoms of eating disorders and suicidal ideation carry substantial ethical, social, and clinical implications. Understanding that individuals who experience symptoms of eating disorders may have a genetic predisposition not only to disordered eating but also to suicidality raises profound questions about autonomy and decision-making in contexts such as assisted dying. In particular, this research intersects with debates around the ethical permissibility of assisted dying for individuals with chronic psychiatric conditions, including eating disorders [73]. If a genetic predisposition links eating disorders with an increased risk for suicidal ideation, it highlights the need for careful clinical assessments that distinguish between transient suicidal impulses influenced by treatable psychiatric or nutritional factors and more enduring expressions of autonomous suicidal intent. Clinically, the findings demand heightened vigilance in suicide risk assessments and the development of tailored interventions that address the unique biological and psychological vulnerabilities contributing to both eating disorders and suicidal ideation.

In conclusion, our study elucidates the phenotypic and genetic associations between eating disorder symptoms and suicidal ideation, suggesting a common latent factor that contributes to the susceptibility of both conditions while also highlighting substantial independent variances. Despite the frequent co-occurrence of eating disorders with other psychiatric conditions, our findings emphasize the specificity of the eating disorders-suicidality relationship, independent of co-occurring psychopathology. These insights necessitate efforts to further characterize the general factor of susceptibility to symptoms of eating disorders and suicidal ideation and explore the degree of genome-wide pleiotropy between these conditions.

## Supporting information

Musial et al. supplementary material Musial et al. supplementary material

## Data Availability

The code for all analyses is available at https://github.com/agmusial/genomic_links_eds_su.

## Supplementary material

The supplementary material for this article can be found at http://doi.org/10.1192/j.eurpsy.2025.25.
