# Supplementary material for: Genomic links between symptoms of eating disorders and suicidal ideation
Source: Eur Psychiatry. 2025 Feb 19;68(1):e38. doi: 10.1192/j.eurpsy.2025.25 (PMC11883781; doi:10.1192/j.eurpsy.2025.25)
Supplement: Musial et al. supplementary material [file S0924933825000252sup001.docx]

Genomic links between symptoms of eating disorders and suicidal ideation.

Agnieszka Musial^1^, Una Foye^1^, Saakshi Kakar^1^, Tom Jewell^2^, Janet Treasure^1,3^, Gursharan Kalsi^1,3^, Iona Smith^1,3^, Laura Meldrum^1,3^, Shannon Bristow^1,3^, Ian Marsh^1,3^, Chelsea Mika Malouf^1,3^, Jahnavi Arora^1,3^, Helena Davies^1,3^, Rina Dutta^4^, Ulrike Schmidt^4^, Gerome Breen^1,3^ & Moritz Herle^1^

^1^Social, Genetic & Developmental Psychiatry Centre, Institute of Psychiatry, Psychology & Neuroscience, King’s College London, London, United Kingdom

^2^Department of Mental Health Nursing, Florence Nightingale Faculty of Nursing, Midwifery & Palliative Care, King’s College London, London, United Kingdom

^3^United Kingdom National Institute for Health and Care Research (NIHR) Maudsley Biomedical Research Centre, South London and Maudsley NHS Foundation Trust, London, United Kingdom

^4^Department of Psychological Medicine, School of Academic Psychiatry, Institute of Psychiatry, Psychology & Neuroscience, King’s College London, London, United Kingdom

Supplementary Notes

Supplementary Note 1

Genotyping and quality control protocol.

All data from GLAD and COPING NBR were genotyped by ThermoFisher on the UK Biobank Axiom Array v1 and v2 across numerous genotyping batches. Genetic data for GLAD and COPING NBR cohorts were merged and restricted to individuals from European ancestries (749,044 SNPs before quality control). Ancestry was determined using GenoPred (<https://opain.github.io/GenoPred/index.html>), by projecting GLAD and COPING individuals on genomic principal components from the 1000 Genomes reference data and assigning individuals a genetic ancestry if they lay< 3 SD from the mean of individuals from that ancestry superpopulation in 1000 Genomes. Quality control was conducted, excluding variants with MAF< 0.01, call rate < 0.95, or which were deviant from Hardy-Weinberg equilibrium (p< 10^-10). Individuals were excluded if they had withdrawn from the study following genotyping, if they were a duplicate of a higher-quality sample (not including known identical twins), if they were known to be mislabelled, if their genotypic sex (males Fx> 0.8, females Fx< 0.5) did not match their sex assigned at birth, if they were outliers on genome-wide heterozygosity (absolute(Fhat)> 0.2), or if they had an excess of relatives (average pi-hat> 3 SD from the mean). Following quality control, 33,635 individuals and 484,182 variants were available for imputation. Imputation was carried out to TopMED Freeze 8, using the dedicated imputation server ( <https://imputation.biodatacatalyst.nhlbi.nih.gov/#!>). Following imputation, data was further restricted to data with MAF >= 0.01 and R2 >= 0.3, leaving 15,009,228 variants for analysis.

Supplementary Note 2

Hypotheses pre-registered with the Open Science Framework (OSF) and deviations from the pre-registration.

1. Symptoms of eating disorders are moderately phenotypically and genetically correlated with self-harm and suicidal ideation.

2. Self-harm and suicidal ideation are strongly correlated with one another, both phenotypically and genetically.

3. Symptoms of eating disorders remain moderately phenotypically and genetically correlated with self-harm and suicidal ideation, irrespective of the co-occurring mood disorders.

4. Results are similar for males and females.

We do not have specific predictions about the latent structure underlying the co-occurrence between eating disorder symptoms, self-harm, and suicidality.

Because the hypotheses were pre-registered prior to accessing the data, we were not able to investigate the phenotypic and aetiological overlap between symptoms of self-harm and suicidality as both conditions were measured by single items as part the same survey (*Thoughts and feelings questionnaire*). We therefore decided to not create self-harm and suicidal ideation-specific subscales and use both construct together in subsequent models. Further, we have planned to use the genome-wide structural equation modelling software (GWSEM 2.0) [1] to run a multivariate genome-wide association study (GWAS) of the latent structure underlying the co-occurrence between symptoms of eating disorders and suicidal ideation. However, due to insufficient sample size, our GWAS did not identify any genome-wide significant variants, therefore precluding us from exploring pleiotropy underlying the association between eating disorder symptoms and suicidal ideation. In order to investigate the genetic overlap between these conditions, instead of running a GWAS of the factor structure, we have instead fitted the phenotypic models and extracted scores for each of the estimated factors, for each individual in our sample, followed by GWAS, SNP heritability and genetic correlation analyses of each individual factor (see *Method*).

Supplementary Figures

|  |
| --- |

Supplementary Figure 1. Exemplar structure of the hierarchical model of eating disorder symptoms and suicidal ideation (panel a), residual model (panel b) and four-factor model (panel c). *Note*. ED= eating disorder; AN= anorexia nervosa; BN= bulimia nervosa; BED= binge-eating disorder; TAF item 1= Have you contemplated harming yourself?; TAF item 2= Many people have thoughts that life is not worth living. Have you felt that way?; TAF item 3= Before the pandemic, had you deliberately harmed yourself, whether or not you meant to end your life?. Items are listed in Supplementary Table 1.

|  |
| --- |

Supplementary Figure 2. Exemplar structure of the hierarchical model of eating disorder symptoms, psychopathology and suicidal ideation (panel a), residual model (panel b), four-factor model (panel c) and three-factor model (panel d). *Note*. ED= eating disorder; AN= anorexia nervosa; BN= bulimia nervosa; BED= binge-eating disorder; TAF item 1= Have you contemplated harming yourself?; TAF item 2= Many people have thoughts that life is not worth living. Have you felt that way?; TAF item 3= Before the pandemic, had you deliberately harmed yourself, whether or not you meant to end your life?. Items are listed in Supplementary Table 1.

|  |
| --- |

Supplementary Figure 3. Correlation matrix (panel a), scree plot (panel b) and results of the confirmatory factor analysis of AN, BN and BED symptom scores, measures of psychopathology and TAF suicidality items (panel c). *Note*. AN= anorexia nervosa; BN= bulimia nervosa; BED= binge-eating disorder; TAF item 1= Have you contemplated harming yourself?; TAF item 2= Many people have thoughts that life is not worth living. Have you felt that way?; TAF item 3= Before the pandemic, had you deliberately harmed yourself, whether or not you meant to end your life?.

|  |
| --- |
| Supplementary Figure 4. Results of the genome-wide association studies of the extracted factor scores from the fitted two-factor (panel a), residual (panel b) and four-factor models (panel c). |

Supplementary Tables

Supplementary Table 1. ED100k items and sub-scales.

| AN items |
| --- |
| \| 1. When you weighed much less than other people thought you ought to weigh or were at your lowest weight, was this due to an illness other than an eating disorder? \| \| --- \| \| 1. During the time when you were at this low weight/BMI, how afraid were you that you might gain weight or become fat? \| \| 1. During the time when you were at this low weight/BMI, how dependent was your self-worth on your body shape or weight? \| \| 1. Did you ever think your low weight/BMI had negative consequences for your health? \| \| 1. During the time when you were at this low weight/BMI, did you still feel fat? \| \| 1. During the time when you were at this low weight/BMI, did you ever experience your body or parts of your body to be larger than they actually were or than other people thought they were? \| |
| BN items |
| 1. Have you ever used any of the following to control your body shape or weight?  - Fasted or did not eat for 8 waking hours or more - Used over the counter or prescription diet pills - Exercised excessively, e.g., felt compelled to exercise, felt uneasy or distressed if unable to exercise - Made yourself vomit - Used laxatives - Used diuretics  1. Excessive exercise  - Have you ever felt compelled to exercise (like you had to do it) to control your body shape or weight? - Have you ever felt uneasy or distressed if unable to exercise? - Have there been times when you declined opportunities to be with friends in order to exercise? - Have you exercised despite an injury or illness that would have prevented others from exercising? - Have there been times when you modified your diet/eating habits if you were unable to exercise for any reason?  1. During the period of time when you were at your lowest weight, did you use any of the following as a way to control your weight or shape?  - Making yourself vomit - Laxatives - Diuretics - Weight loss pills - Excessive exercise - Fasting - Other methods - None of the above  1. Have you ever used any of the following to compensate for episodes of binge eating or overeating?  - Making yourself vomit - Laxatives - Diuretics - Weight loss pills - Excessive exercise - Fasting - Other methods - None of the above |
| BED items |
| 1. Have you ever had regular episodes of overeating or eating binges when you ate what most people would regard as an unusually large amount of food in a short period of time? (for example, in a 2-hour period) 2. When you were having regularly occurring episodes of binge eating or overeating, did you feel that your eating was out of control such that you felt you could not stop eating, or that you could not control what or how much you were eating? 3. Do you/did you feel distressed about your episodes of over eating? 4. During the time when you were binge eating, how dependent was your self-worth on your body shape or weight? 5. Did you experience regular episodes of binge eating while at your lowest weight? 6. How distressed did binge eating usually make you feel? 7. During eating binges, did you:  - Eat much more rapidly than usual - Eat until you felt uncomfortably full - Eat large amounts of food when you didn’t feel physically hungry - Eat alone because you were embarrassed by what/how much you were eating - Feel ashamed/disgusted with yourself or depressed or very guilty after overeating - Feel like you had no control over your eating, e.g., not being able to stop eating, feeling compelled to eat or going back and forth for more food - Make yourself vomit as a means to control your weight and shape |

*Note*. Binary items, such as *During eating binges, did you feel ashamed/disgusted with yourself, depressed, or very guilty after overeating?* were summed up to create BN and BED quantitative subscales related to weight and shape control, compensatory behaviours, excessive exercise and bingeing emotions/behaviours, where higher scores reflected more severe symptoms. We subsequently created scales specific to AN, BN and BED by summing item scores. These scales are used in the majority of fitted models, except for the four-factor model that distinguished between restricting, purging and bingeing types of eating disorder behaviours. The four-factor model included AN-related items as manifest variables loading onto the restricting factor, BN-related items as manifest variables loading onto the purging factor and BED-related items loading onto the bingeing factor; AN= anorexia nervosa; BN= bulimia nervosa; BED= binge-eating disorder.

Supplementary Table 2. Model fit indices of the latent structures underlying the co-occurrence between symptoms of eating disorders and suicidal ideation .

| Model | CFI | TLI | AIC | BIC | RMSEA (95% CIs) |
| --- | --- | --- | --- | --- | --- |
| EFA-based | | | | | |
| Two-factor eating disorders & suicidal ideation | 0.982 | 0.965 | 116719.788 | 116855.817 | 0.074 (0.068, 0.080) |
| Two-factor eating disorders & psychopathology & suicidal ideation | 0.820 | 0.734 | 162692.794 | 162872.935 | 0.190 (0.186, 0.194) |
| Theoretical | | | | | |
| Hierarchical model eating disorders & suicidal ideation | 0.982 | 0.961 | 393016.955 | 393184.465 | 0.079 (0.075, 0.082) |
| Residual model eating disorders & suicidal ideation | 0.999 | 0.994 | 391723.046 | 391924.058 | 0.032 (0.027, 0.037) |
| Hierarchical model eating disorders & psychopathology & suicidal ideation | 0.825 | 0.728 | 547117.079 | 547335.971 | 0.194 (0.192, 0.196) |
| Residual model eating disorders & psychopathology & suicidal ideation | 0.994 | 0.986 | 525303.350 | 525572.755 | 0.044 (0.042, 0.047) |
| Four-factor restricting & purging & bingeing & suicidal ideation | 0.962 | 0.956 | 664093.358 | 664646.143 | 0.030 (0.029, 0.031) |
| Three-factor eating disorders & suicidal ideation & affective disorders | 0.976 | 0.961 | 527567.744 | 527795.055 | 0.073 (0.071, 0.075) |

*Note*. CFI= comparative fit index; TLI= Tucker-Lewis Index; AIC= Akaike information criterion; BIC= Bayesian information criterion; RMSEA= Root Mean Square Error of Approximation; CIs= confidence intervals.

Supplementary Table 3. Results of the SNP heritability and genetic correlation analyses.

| Factor | SNP h^2^ (GCTA-GREML) |
| --- | --- |
| EFA-based | |
| Suicidal ideation | 0.081 (0.026) |
| Eating disorders | 0.103 (0.026) |
| Theoretical | |
| General susceptibility | 0.10 (0.026) |
| Residual suicidal ideation | 0.053 (0.025) |
| Residual eating disorders | 0.086 (0.026) |
| Restricting | 0.089 (0.026) |
| Purging | 0.116 (0.026) |
| Bingeing | 0.118 (0.026) |
| Suicidal ideation | 0.084 (0.026) |
|  | |
| Factor | SNP rG (GCTA-GREML) |
| EFA-based | |
| Suicidal ideation & Eating disorders | 0.49 (0.16) |
| Theoretical | |
| Residual suicidal ideation & Residual eating disorders | -0.397 (0.233) |
| Restricting & Suicidal ideation | 0.793 (0.147) |
| Purging & Suicidal ideation | 0.560 (0.150) |
| Bingeing & Suicidal ideation | 0.515 (0.140) |
| Restricting & Purging | 0.960 (0.033) |
| Restricting & Bingeing | 0.839 (0.065) |
| Purging & Bingeing | 0.823 (0.063) |

*Note.* EFA= exploratory factor analysis; GCTA-GREML= genome-wide complex trait analysis- genome-based restricted maximum likelihood; SNP h^2^= SNP heritability; rG= genetic correlation; SE= standard error.

Supplementary Table 4. Results of the sensitivity SNP heritability and genetic correlation analyses, using a sub-sample without individuals diagnosed with MDD/GAD.

| Factor | SNP h^2^ (GCTA-GREML) |
| --- | --- |
| EFA-based | |
| Suicidal ideation | 0.002 (0.059) |
| Eating disorders | 0.153 (0.061) |
| Theoretical | |
| General susceptibility | 0.094 (0.061) |
| Residual suicidal ideation | 0.085 (0.059) |
| Residual eating disorders | 0.070 (0.058) |
| Restricting | 0.217 (0.060) |
| Purging | 0.213 (0.060) |
| Bingeing | 0.172 (0.061) |
| Suicidal ideation | 0.000 (0.058) |
|  | |
| Factor | SNP rG (GCTA-GREML) |
| EFA-based | |
| Suicidal ideation & Eating disorders | 1.00 (2.209) |
| Theoretical | |
| Residual suicidal ideation & Residual eating disorders | 0.418 (0.694) |
| Restricting & Suicidal ideation | 1.00 (2.821) |
| Purging & Suicidal ideation | 1.00 (4.471) |
| Bingeing & Suicidal ideation | -1.00 (49.005) |
| Restricting & Purging | 0.965 (0.038) |
| Restricting & Bingeing | 0.698 (0.107) |
| Purging & Bingeing | 0.957 (0.075) |

*Note.* MDD= major depressive disorder; GAD= generalized anxiety disorder; EFA= exploratory factor analysis; GCTA-GREML= genome-wide complex trait analysis- genome-based restricted maximum likelihood; SNP h^2^= SNP heritability; rG= genetic correlation; SE= standard error; due to low power several genetic correlation analyses did not converge.

Supplementary Table 5. Results of the sensitivity SNP heritability and genetic correlation analyses, using a sub-sample of males only.

| Factor | SNP h^2^ (GCTA-GREML) |
| --- | --- |
| EFA-based | |
| Suicidal ideation | 0.007 (0.078) |
| Eating disorders | 0.150 (0.081) |
| Theoretical | |
| General susceptibility | 0.028 (0.079) |
| Residual suicidal ideation | 0.027 (0.078) |
| Residual eating disorders | 0.224 (0.083) |
| Restricting | 0.112 (0.076) |
| Purging | 0.181 (0.081) |
| Bingeing | 0.037 (0.075) |
| Suicidal ideation | 0.002 (0.078) |
|  | |
| Factor | SNP rG (GCTA-GREML) |
| EFA-based | |
| Suicidal ideation & Eating disorders | 0.479 (2.556) |
| Theoretical | |
| Residual suicidal ideation & Residual eating disorders | -0.103 (0.724) |
| Restricting & Suicidal ideation | 1.00 (9.191) |
| Purging & Suicidal ideation | 1.00 (6.086) |
| Bingeing & Suicidal ideation | 1.00 (11.558) |
| Restricting & Purging | 1.00 (0.064) |
| Restricting & Bingeing | 1.00 (0.457) |
| Purging & Bingeing | 1.00 (0.463) |

*Note.* EFA= exploratory factor analysis; GCTA-GREML= genome-wide complex trait analysis- genome-based restricted maximum likelihood; SNP h^2^= SNP heritability; rG= genetic correlation; SE= standard error; due to low power several genetic correlation analyses did not converge.

Supplementary Table 6. Results of the sensitivity SNP heritability and genetic correlation analyses, using a sub-sample of females only.

| Factor | SNP h^2^ (GCTA-GREML) |
| --- | --- |
| EFA-based | |
| Suicidal ideation | 0.131 (0.036) |
| Eating disorders | 0.095 (0.036) |
| Theoretical | |
| General susceptibility | 0.124 (0.036) |
| Residual suicidal ideation | 0.077 (0.035) |
| Residual eating disorders | 0.072 (0.035) |
| Restricting | 0.094 (0.035) |
| Purging | 0.123 (0.036) |
| Bingeing | 0.127 (0.036) |
| Suicidal ideation | 0.134 (0.036) |
|  | |
| Factor | SNP rG (GCTA-GREML) |
| EFA-based | |
| Suicidal ideation & Eating disorders | 0.492 (0.183) |
| Theoretical | |
| Residual suicidal ideation & Residual eating disorders | -0.479 (0.284) |
| Restricting & Suicidal ideation | 0.768 (0.160) |
| Purging & Suicidal ideation | 0.512 (0.161) |
| Bingeing & Suicidal ideation | 0.526 (0.148) |
| Restricting & Purging | 0.950 (0.044) |
| Restricting & Bingeing | 0.790 (0.092) |
| Purging & Bingeing | 0.798 (0.085) |

*Note.* EFA= exploratory factor analysis; GCTA-GREML= genome-wide complex trait analysis- genome-based restricted maximum likelihood; SNP h^2^= SNP heritability; rG= genetic correlation; SE= standard error; due to low power several genetic correlation analyses did not converge.
